# Identification and Quantification of Gluconic, Metasaccharinic, Glyceric, Formic, and Acetic Acids as Novel Glucose Degradation Products in Peritoneal Dialysis Fluids

**DOI:** 10.1021/acsomega.6c02048

**Published:** 2026-06-04

**Authors:** Sabrina Gensberger-Reigl, Jinan Awada, Vera L. Rodrigues Guimarães Abreu, Ingrid Weigel, Dimitra Tousi, Elisa Gardill, Pascal Mathis, Monika Pischetsrieder

**Affiliations:** 1 Chair of Food Chemistry, Department Chemistry and Pharmacy, Friedrich-Alexander-Universität Erlangen-Nürnberg (FAU), Nikolaus-Fiebiger-Str. 10, Erlangen 91058, Germany; 2 FAU NeW - Research Center New Bioactive Compounds, Friedrich-Alexander-Universität Erlangen-Nürnberg (FAU), Nikolaus-Fiebiger-Str. 10, Erlangen 91058, Germany; 3 348325 Fresenius Medical Care Deutschland GmbH, Frankfurter Straße 6-8, St. Wendel 66606, Germany

## Abstract

Peritoneal dialysis (PD) fluids often contain glucose as the osmotic agent. Heat sterilization ensures their microbiological safety, but the heat impact degrades glucose. The altered composition and reduced biocompatibility of the fluids limit the long-term use of PD. A key indicator of glucose degradation is a decrease in pH poststerilization suggesting that carboxylic glucose degradation products (carboxyl GDPs) were formed. The present study employed a precursor ion mass spectrometry screening using 3-nitrophenylhydrazine derivatization to investigate the presumed carboxylic products. Based on enhanced product ion spectra, structures were suggested, which were confirmed by comparing the chromatographic and mass spectrometric properties of the unknown signals to reference compounds. Thus, five carboxylic acids were identified in commercial PD fluids, namely, gluconic, metasaccharinic, glyceric, formic, and acetic acids. The new carboxyl GDPs were quantified in the PD fluids showing 47.8–62.6 μM gluconic, 125.8–180.7 μM metasaccharinic, 3.2–6.6 μM glyceric, 28.4–37.3 μM acetic, and 60.1–92.3 μM formic acids. The total amount of the newly identified acids ranging from 287.3 to 373.3 μM explains almost completely the poststerilization pH decrease of the PD fluids. The present findings show that heat sterilization induces the formation of carboxylic glucose degradation products in PD fluids.

## Graphical abstract

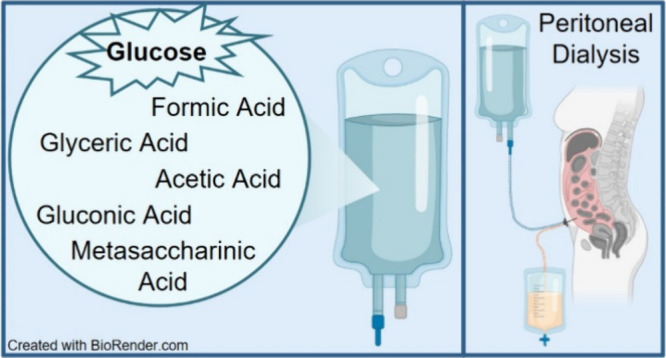

## Introduction

Peritoneal dialysis (PD) is a renal replacement therapy using the patient’s peritoneum as a semipermeable membrane to pass water and uremic toxins from the blood into an osmotically active PD fluid.^1^ PD fluids contain a buffering substance and electrolytes such as calcium, magnesium, sodium, and chloride and commonly utilize highly concentrated glucose solutions as the osmotic agent. PD fluids are heat-sterilized during production to ensure the microbiological safety. The heat stress leads to a partial breakdown of glucose and, thus, to the formation of glucose degradation products (GDPs).^2,3^ Consequently, commercially available glucose-based PD fluids contain monocarbonyl GDPs, such as 5-hydroxymethylfurfural (5-HMF), furfural, acetaldehyde, and formaldehyde.^4–6^ Additionally, α-dicarbonyl GDPs like glucosone, 3-deoxyglucosone (3-DG), 3-deoxygalactosone (3-DGal), 3,4-dideoxyglucosone-3-ene (3,4-DGE), glyoxal, and methylglyoxal were identified and quantified in different commercial PD fluids.^5,7,8^ GDPs may harm the peritoneal membrane, for example, by their cytotoxic activity or the enhanced formation of advanced glycation end products (AGEs). The effects impair the biocompatibility of PD fluids and limit a long-term therapy.^9–12^ To counter these detrimental consequences, different methods have been developed to analyze monocarbonyl and α-dicarbonyl GDPs and control their formation in PD fluids.^6,13–17^

To date, research on glucose degradation during heat sterilization of PD fluids has focused on monocarbonyl and α-dicarbonyl compounds. However, these may not be the only degradation products formed from glucose; moreover, different GDPs can contribute differently to adverse effects. Therefore, it is important to obtain a comprehensive overview of all GDP structures present in PD fluids, which is the basis to successfully assess their biological and toxicological effects. For example, 3,4-DGE, a rather low abundant GDP in PD fluids, has shown a major impact on cell viability *in vitro* compared to higher abundant GDPs such as 3-DG or glucosone.^18,19^ A decrease of the pH value after heat sterilization was observed in PD fluids even though the solutions are mostly lactate-buffered.^15^ This points to the formation of carboxylic acids. The formation of carboxylic acids during glucose degradation has been described before.^20–22^ However, their presence in PD fluids has not been detected so far, most likely, because they are not covered by the analytical methods currently applied for the analysis of mono- and α-dicarbonyl compounds. Therefore, a targeted suspect screening of GDPs with carboxyl groups (carboxyl GDPs) was applied using precursor ion mass spectrometry. For this purpose, carboxylic acids were reacted with 3-nitrophenylhydrazine (3-NPH) and analyzed by ultrahigh-performance liquid chromatography hyphenated diode array detection (UHPLC–DAD) and tandem mass spectrometry (UHPLC–MS/MS).

## Experimental Setup

### Chemicals, Reagents, Reference Standards, and Samples

All chemicals, reagents, and standards were purchased from Merck Sigma-Aldrich (Steinheim, Germany) and were at least of analytical grade unless noted otherwise. ROTISOLV acetonitrile (LC-MS grade) and calcium oxide (≥96%) were obtained from Carl Roth (Karlsruhe, Germany). Formic acid (≥99%, LC-MS grade), acetic acid (99–100%), ethanol, ethyl acetate, and sodium chloride were obtained from VWR (Darmstadt, Germany). Dimethyl sulfoxide-*d*_6_ (DMSO-*d*_6_) was supplied by Deutero (Kastellaun, Germany) and tetramethyl silane (NMR grade 99.9+%) by Acros Organics (Thermo Fisher Scientific, Dreieich, Germany). Ultrapure water was taken from a Milli-Q Reference A+ system (Merck, Darmstadt, Germany). Isosaccharinic-1,4-lactone was purchased from Biosynth (Berlin, Germany). Gluconate-1-^13^C sodium was purchased from Hoelzel Diagnostika Handels (Köln, Germany) and ^13^C-glyceric acid calcium salt dihydrate from Euriso-Top (Saarbrücken, Germany). The commercial PD fluids contained 4.25% glucose, 35 mM lactate, 134 mM sodium, 1.75 mM calcium, 0.5 mM magnesium, and 104 mM chloride. Carboxyl GDPs were quantified in five different batches of PD fluids. Unheated PD fluids were prepared to match the composition of commercial solutions using European Pharmacopeia-grade glucose monohydrate (48 g/L), sodium lactate (4.0 g/L), sodium chloride (5.7 g/L), calcium chloride dihydrate (257 mg/L), magnesium chloride hexahydrate (102 g/L), and ultrapure water.

### Instrumentation

NMR spectra were recorded by an Avance 600 system (Bruker Biospin, Rheinstetten, Germany). Signals were assigned on the basis of one-dimensional 1H and distorsionless enhancement by polarization transfer quantum coherence (DEPTQ) as well as two-dimensional 1H,1H-correlated spectroscopy (COSY), heteronuclear single quantum coherence (HSQC), and heteronuclear multiple bond correlation (HMBC). To identify acidic protons, H/D exchange experiments were performed. The UHPLC experiments were carried out on Dionex UltiMate 3000RS or Vanquish Flex instruments. Both systems (Thermo Fisher Scientific, Dreieich, Germany) consisted of a pump with degasser, autosampler, and column compartment each. Chromatographic separation was achieved on a Kinetex Core–Shell C18 column (1.7 μm particle size, 2.1 × 150 mm, Phenomenex, Aschaffenburg, Germany). The Vanquish Flex UHPLC system was equipped with DAD and controlled by Chromeleon 7.2 software. The Dionex UltiMate UHPLC was connected to a mass spectrometer 4000 QTrap equipped with an electrospray ionization (ESI) source (Sciex, Darmstadt, Germany). The ESI–MS was run in negative mode, operated at 450 °C with a voltage of −4000 V, declustering potential of −60 V, dry gas of 35 psi, and nebulizer gas of 35 psi. Nitrogen was used for collision-induced dissociation. For precursor ion scans, *m*/*z* 137 was used as the fragment mass. Full mass scans and product ion spectra were obtained in QTrap-enhanced mode (enhanced mass scan, EMS; enhanced product ion spectra, EPI). Analyst 1.6.2 software was used for data acquisition and processing. High-resolution mass spectrometry experiments were carried out on timsTOF Pro 2 instrument equipped with an Apollo II ion source coupled to elute UHPLC system (Bruker, Bremen, Germany). The instrument was run in negative ionization mode, operated at 220 °C with a voltage of −4200 V and dry gas 10.0 l/min. Data Analysis 6.1 software was used for data evaluation. For method validation and subsequent quantification, a Shimadzu Nexera LC-40 system consisting of a dual pump, autosampler, column compartment, and diode array detector was coupled to a Sciex 6500+ QTrap mass spectrometer (Sciex, Darmstadt, Germany). Sciex OS 3.4 software was used for data analysis. The 6500+ QTrap mass spectrometer was run in negative multiple reaction monitoring (MRM) mode and operated at 550 °C with a voltage of −3500 V, dry gas of 85 psi, and nebulizer gas of 55 psi. Declustering, cell entrance, and cell exit potentials as well as collision energies were optimized for each transition (Supporting Information Table S1).

### pH Measurements before and after Sterilization

The pH values of four PD fluids were determined in duplicate before and after heat sterilization. During the measurements by a SevenExcellence Multiparameter system (Mettler Toledo, Gießen, Germany), the samples were kept at 25.0 ± 0.2 °C in a water bath. A one-sided paired *t*-test was performed to evaluate significant changes in pH before and after sterilization.

### Synthesis of Reference Compounds

Two reference compounds were commercially not available and were synthesized as follows.

#### 5-(1,2-Dihydroxyethyl)-3-hydroxydihydrofuran-2­(3*H*)-one (Metasaccharinic Acid γ-Lactone)

Metasaccharinic acid γ-lactone was synthesized according to Chiku et al. with some modifications.^23^ Briefly, 0.9 g of turanose was dissolved in 50 mL of sodium phosphate buffer (100 mM, pH 7.45). The solution (20 mL) was heated for 14 h at 90 °C. After cooling to room temperature, 5.2 g of Amberlite IRN-150 ion exchanger was added and the solution was stirred for 40 min. Then, the reaction mixture was filtered, freeze-dried, and purified by flash chromatography on silica 60 (0.2–0.5 mm, Merck, Darmstadt, Germany) using a mixture of acetonitrile and water (95:5). Chromatographic separation was controlled by thin layer chromatography [TLC; eluent: 2-propanol/water/25% ammonium hydroxide (14:3:3)] using sulfuric acid/*p*-anisaldehyde/acetic acid/ethanol (13:13:5:478) for derivatization. For further purification, the synthesis product was dissolved in 2 mL of sodium hydroxide (pH 11) and an aliquot of 1 mL was loaded onto the anion exchanger (Strata-X-AW 30 mg/mL sorbent mass, Phenomenex, Aschaffenburg, Germany), which had been activated with methanol and equilibrated with water. After the sample was washed with 1 mL of ammonium acetate (25 mM) and then with 1 mL of methanol, the synthesis product was eluted in two steps, each time with 500 μL of formic acid (5%) in methanol, and then lyophilized to yield 13 mg. The product was characterized by NMR (atom numbering refers to Scheme 1). For this, 6 mg of the synthesis product was dissolved in 0.75 mL of DMSO-*d*_6_; trimethyl silane was used as an internal standard. NMR: COSY (DMSO-*d*_6_): δ 1.56/1.83 (2H, H-3), 3.21 (1H, H-5), 3.33/3.55 (2H, H-6), 3.48 (1H, H-4), 3.78 (1H, H-2), 4.30 (1H, OH-6), 4.50 (1H, OH-2), 6.03 (1H, OH-5); DEPTQ (DMSO-*d*_6_): δ 39.2 (C-3), 64.2 (C-6), 70.3 (C-4), 70.5 (C-2), 75.6 (C-5), 179.5 (C-1).

**Scheme 1:**
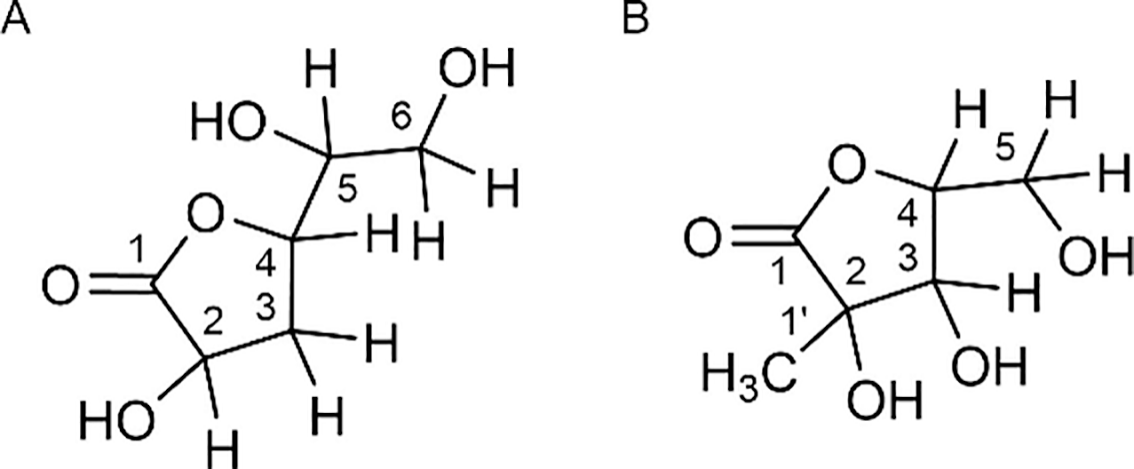
γ-Lactones of (A) Metasaccharinic Acid and (B) Saccharinic Acid

#### 3,4-Dihydroxy-5-(hydroxymethyl)-3-methyldihydrofuran-2­(3*H*)-one (Saccharinic Acid γ-Lactone)

Saccharinic acid γ-lactone was synthesized according to Hotchkiss et al.^24^ and Voigt.^25^ Briefly, glucose (3 g) was suspended in 5 mL of ethanol and 1 mL of acetic acid (99%). After adding 3 mL of dimethylamine in ethanol (33%), the solution was heated under reflux for 1.5 h at 80 °C. The reaction mixture was cooled down and evaporated until a highly viscous, oily, dark brown suspension remained. It was mixed with 150 mL of water and 6 g of calcium oxide and stirred for 24 h at 70 °C. After adding 7 g of oxalic acid dihydrate, the suspension was filtered, aided by Celite 535, and purified using an Amberlite IR-120 ion exchanger, which was activated with water. The synthesis product was eluted with water. The eluate was evaporated until dryness and resolved in 50 mL of ultrapure water. A second purification step was performed by flash chromatography. After adding 50 mL of a mixture (1:1) of ethyl acetate and cyclohexane and 10 g of silica, the synthesis product was evaporated until dryness. The synthesis product was then loaded onto a flash chromatography column filled with silica and a mixture (1:1) of ethyl acetate and cyclohexane. Elution was performed with 100% ethyl acetate and controlled by TLC. Fractions in which TLC indicated the presence of saccharinic acid γ-lactone were combined and concentrated under reduced pressure. The synthesis product was recrystallized from a 1:1 mixture of ethyl acetate and cyclohexane and lyophilized to yield 121 mg of saccharinic acid γ-lactone. The product was characterized by NMR (atom numbering refers to Scheme 1). For this, 15 mg of the synthesis product was dissolved in 0.75 mL of DMSO-*d*_6_; trimethyl silane was used as an internal standard. 1H NMR (DMSO-*d*_6_): δ 1.25 (3H, H-1′), 3.52 (1H, H-5a), 3.69 (1H, H-5b), 3.78 (1H, H-3), 4.16 (1H, H-4), 5.00 (1H, OH-5), 5.42 (1H, OH-3), 5.69 (1H, OH-2). DETPQ (DMSO-*d*_6_): δ 21.3 (C-1’), 60.0 (C-5), 72.4 (C-2), 72.5 (C-3), 83.2 (C-4), 176.6 (C-1).

### Derivatization Procedure

The derivatization solutions were prepared according to Han et al.^26^ Briefly, 3-NPH hydrochloride was dissolved in 50% acetonitrile to a concentration of 200 mM; 1-ethyl-3-(3-(dimethylamino)­propyl)­carbodiimide hydrochloride (EDC, 120 mM) was dissolved in a mixture of 50% acetonitrile and 6% pyridine. The compounds of interest were derivatized according to Hofstetter et al.^27^ Briefly, 40 μL of each sample was mixed thoroughly with 20 μL of 3-NPH hydrochloride and 20 μL of EDC solution and incubated at 40 °C for 30 min (qualitative analysis) or 60 min (quantitative analysis). Then, 920 μL of acetonitrile (50%) was added before each solution was stirred and filtered (PVDF membrane, 0.22 μm pore size, ROTILABO, Carl Roth).

### Chromatographic Separation

Separation was achieved by UHPLC–DAD–MS/MS applying the following parameters: eluent A, 0.1% formic acid in ultrapure water; eluent B, 0.1% formic acid in acetonitrile; flow rate, 350 μL/min; gradient elution time (min)/% B, −3.0/13, 3.0/13, 9.5/55, 11/95, 13/95; column compartment temperature 35 °C. Aliquots of 1 μL and 5 μL for EPI experiments were injected. Data acquisition started at 0.0 min, and chromatograms were monitored at 254 nm. DAD spectra were recorded from 190 to 700 nm.

### Enzymatic Analysis of Formic Acid and Acetic Acid

Formic acid and acetic acid were enzymatically detected by the commercial test kits Enzytec Liquid Formic Acid and Enzytec Acetic Acid (R-Biopharm, Darmstadt, Germany). To assess the recovery rates of the enzymatic assays, an unheated PD solution was spiked with 122.7 μM formic acid and 43.6 μM acetic acid and analyzed in the same manner as the PD fluids. The recovery rate was calculated as follows: (analyzed analyte concentration)/(spiked analyte concentration) × 100%. The recovery rate for formic acid was 95.0% and 100.9% for acetic acid, indicating good assay performance.

### Optimization of the Derivatization Conditions and Stability of Hydrazide Derivatives

First, the impact of derivatization time was assessed at 15, 30, 60, and 90 min in standard solutions and in PD matrix at three different concentration levels for gluconic, glyceric, and metasaccharinic acids (10, 100, and 200 μM, respectively). To assess the stability of hydrazide derivatives, standard solutions of gluconic, glyceric, and metasaccharinic acids were derivatized for 60 min and subjected to repeated analysis at hourly intervals over a 6 h period. In parallel, an unheated PD matrix (containing 35 mM lactate, 134 mM sodium, 1.75 mM calcium, 0.5 mM magnesium, and 104 mM chloride) spiked with the same three acids was analyzed under identical conditions. Each experiment was conducted at three concentration levels: 10, 100, and 200 μM in triplicates.

### UHPLC–MS/MS Method Validation and Quantification

The validation process included the parameters linearity, limit of detection (LOD), limit of quantification (LOQ), and trueness as assessed through intraday precision, interday precision, and recovery rates. Six-point calibration curves were prepared for gluconic, metasaccharinic, and glyceric acids covering a concentration range from 1.0 to 30.1 μM for gluconic, 1.0 μM to 148.8 μM for metasaccharinic, and 0.5 to 10.0 μM for glyceric acids. Each calibration point was analyzed in triplicate, and each set of triplicates was analyzed on three different days. The curves were obtained by plotting the quotient of the peak area of the quantifier for the respective analyte and the quantifier for the corresponding labeled standard. Since no labeled metasaccharinic standard was available, gluconic-1-^13^C acid was used in this case. Linear regression analysis was performed with a minimal coefficient of determination (*R* ^2^) of 0.990. Recovery experiments were conducted by spiking a PD model matrix with three different concentration levels. The concentration levels were analyzed in triplicate, and each set of triplicates was analyzed on three different days to evaluate the recovery rates. The recovery rates were calculated as follows: (analyzed analyte concentration)/(spiked analyte concentration) × 100%. Precision was assessed through reproducibility (interday) and intermediate precision (intraday) expressed as relative standard deviation (RSD). LOD and LOQ were determined based on the signal-to-noise ratio (S/N). An S/N of 3:1 was required for LOD, while an S/N of 10:1 was set as the criterion for LOQ.

### pH Calculation after Sterilization

The resulting pH of the lactate-buffered PD solution after sterilization was calculated using a proton balance approach. The initial conditions (pH 5.5, 35 mM lactate) were used as a reference state. For each acid formed during sterilization, the degree of dissociation at the given pH 5.5 (initial conditions) was determined using the Henderson–Hasselbalch equation.^28^ The protons released by all acids were balanced against the protons absorbed by the buffer and the change in free hydrogen ion concentration. This relationship was expressed in pH and solved iteratively using Excel’s Goal Seek function, adjusting the pH until the net proton balance equaled zero. The measured concentrations of added acids and their p*K*_a_ values (gluconic acid: p*K*_a_ 3.86,^29^ metasaccharinic acid: p*K*_a_ 3.59,^30^ glyceric acid: p*K*_a_ 3.52,^29^ acetic acid: p*K*_a_ 4.76,^31^ and formic acid: p*K*_a_ 3.75 ^31^) were provided as input.

### Statistics

Microsoft Excel 2021 was used for the data analysis.

## Results and Discussion

The present study investigated the impact of heat sterilization on the pH values of PD fluids and screened for novel GDPs with carboxylic moieties.

### pH Changes in PD Fluids after Sterilization

The average pH of unheated PD fluids was 5.53 ± 0.03, while heat-sterilized PD fluids showed an average pH of 5.38 ± 0.01. Thus, the heat impact significantly decreased the pH (*p* < 0.001) despite the presence of the lactate buffer. These findings are in line with a previous study by Erixon et al., who also noted a decrease in pH after heat sterilization.^15^ A possible explanation could be GDPs with carboxyl groups, which may be formed during heat sterilization of glucose-based PD fluids additionally to the well-documented mono- and α-dicarbonyls.

### Screening for Glucose Degradation Products with Carboxyl Groups

Because it was hypothesized that carboxylic acids represent novel GDPs in sterilized PD fluids, targeted screening for carboxyl GDPs was carried out. A major challenge was the low concentration of GDPs in PD fluids in comparison to the excess of glucose and lactate. Structural similarities between glucose, lactate, and the GDPs further complicated the analysis. Since the target analytes lack a chromophore or fluorophore, their detection is challenging. In addition, their high polarity leads to poor retention on reversed-phase stationary phases, which often results in additional difficulties with chromatographic separation.^32^ Therefore, a derivatization step by 3-NPH was used in this study prior to UHPLC–DAD–MS/MS analysis.^26,27^

In contrast to the parent compounds, the formed 3-NP hydrazides were separable by reversed-phase chromatography, UV-active, and easily ionized by ESI. Thus, a UHPLC method was developed to enable baseline separation of most signals detected by UHPLC–DAD (Figure 1A). The main fluid components lactate and glucose and other GDPs such as α-dicarbonyls or monocarbonyls can react also with 3-NPH to form hydrazide (in the case of lactate) or hydrazone derivatives (in the case of glucose, mono-, and α-dicarbonyls).

**Figure 1:**
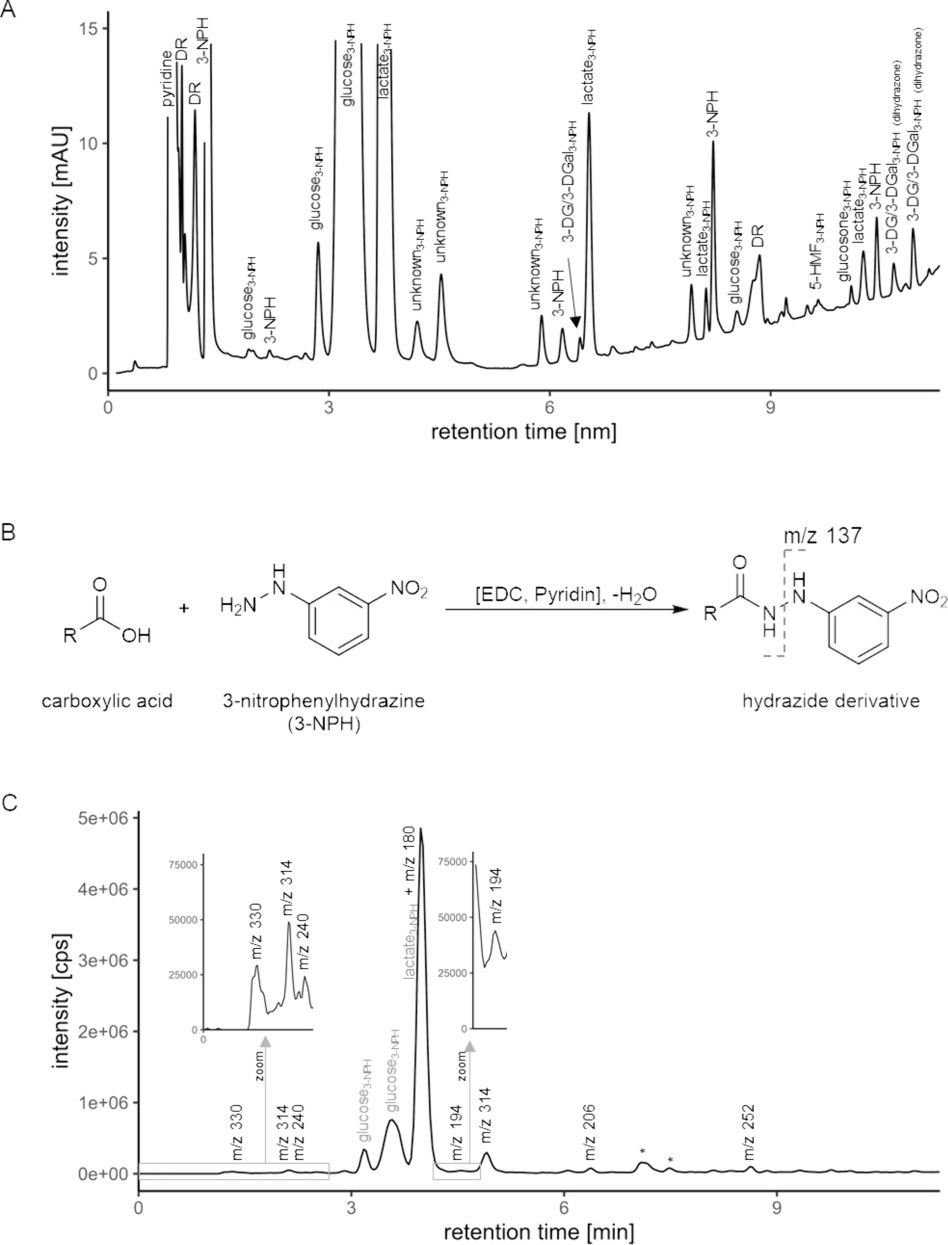
(A) UHPLC–DAD chromatogram of a representative PD fluid after derivatization with 3-NPH (DR, derivatizing reagent; 3-NPH, 3-nitrophenylhydrazine). (B) Conversion of carboxylic acids to hydrazide derivatives results in the introduction of a marker fragment ion *m*/*z* 137. (C) Total ion chromatogram from precursor ion mass spectrometry targeting precursors that yield a fragment at *m*/*z* 137. Signals marked with an asterisk could not be confirmed by full mass scans.

The UHPLC–DAD analysis encountered considerable difficulties in assigning the UV-active signals. The derivatizing reagent itself led to an intense signal, and the major matrix components, i.e., lactate and glucose, produced strong and sometimes multiple signals after derivatization with 3-NPH. Known GDPs, including glucosone, 3-DG or 3-DGal, and 5-HMF, also resulted in intense UV-active signals. Hence, the high matrix load resulted in a very complex chromatogram (Figure 1A) and hampered the detection and identification of novel carboxyl GDPs. Besides, UV/vis detection was not specific enough to distinguish between derivatives of carboxyl GDPs, mono- and α-dicarbonyl GDPs, or matrix components. In addition, it was expected that UV/vis detection would not be sensitive enough to cover all carboxyl GDPs.

MS/MS analysis in contrast should allow a selective and sensitive detection of the signals of interest. Therefore, the samples were subjected to precursor ion MS to selectively detect low-concentrated carboxyl GDPs despite the high abundance of matrix components and other GDPs. Collision-induced dissociation of 3-NP hydrazide derivatives resulted in the specific and characteristic fragment *m*/*z* 137 (Figure 1B). Precursor ion scan MS recorded all parent ions yielding a fragment *m*/*z* 137 without knowing the precursor mass. As expected, the lactate derivative gave the most intense signal (Figure 1C). Additionally, the precursor ion scans detected two signals for glucose because of its excess in the PD fluids. Eight signals in total were identified in the precursor ion scans that potentially originated from carboxylic acids (Table 1). These precursors were detected in all of five tested commercial PD fluids. Since the signal-to-noise ratio in precursor ion scans was rather low (seven for *m*/*z* 314 and three for *m*/*z* 330 and *m*/*z* 240), full mass scans were run to confirm the *m*/*z* values of the precursors. To improve S/N, enhanced full mass scans were conducted using the instrument’s linear ion trap to enrich ions prior to detection. This approach improved the S/N to 83 for *m*/*z* 314, 11 for *m*/*z* 330, and 9 for *m*/*z* 240, allowing confirmation of precursor masses in all five commercial PD fluids. Enhanced product ion scans were employed for compound characterization, enabling ion enrichment and thereby improving S/N.

**Table 1:** Retention times, Measured *m/z*, Molecular Mass of 3-NPH Derivatives, and Molecular Mass of Potential GDPs with a Carboxyl Group in PD Fluids Identified by Precursor Ion Mass Spectrometry

retention time [min]	*m*/*z* measured [M – H^+^]^−^ [Da]	molecular mass of the 3-NPH derivatives [g/mol]	molecular mass of putative carboxylic acid [g/mol]
1.6	330	331	196
2.1	314	315	180
2.3	240	241	106
3.8	180	181	46
4.4	194	195	60
4.7	314	315	180
6.8	206	207	72
8.5	252	253	118

### Identification and Characterization of Unknown Carboxyl GDPs

Carboxylic acids react with 3-NPH to yield a hydrazide moiety, which leads to a mass increase of 135 g/mol. Consequently, the molecular masses of putative carboxylic acids could be calculated on the basis of their precursor masses (Table 1).

Thus, the precursor *m*/*z* 330, eluting at 1.6 min, may refer to a carboxylic acid with a molecular mass of 196 g/mol. This compound was assumed to be gluconic acid, which can be formed by the oxidation of glucose. To prove this hypothesis, commercial gluconic acid was reacted with 3-NPH under the same conditions. UHPLC–MS/MS analysis of the resulting derivative showed the same retention time and product ion spectrum as the unknown compound (Figure 2A/B). Coinjection of the gluconic acid standard with a PD solution led to an increased signal at 1.6 min, without any changes in retention time or in the MS and MS/MS spectra. High-resolution mass spectrometry resulted in *m*/*z* 330.0947 ([M – H^+^]^−^, C_12_H_17_N_3_O_8_, calculated *m*/*z* 330.0932, mass error: 4.5 ppm). Thus, gluconic acid could be unequivocally identified in PD fluids.

**Figure 2:**
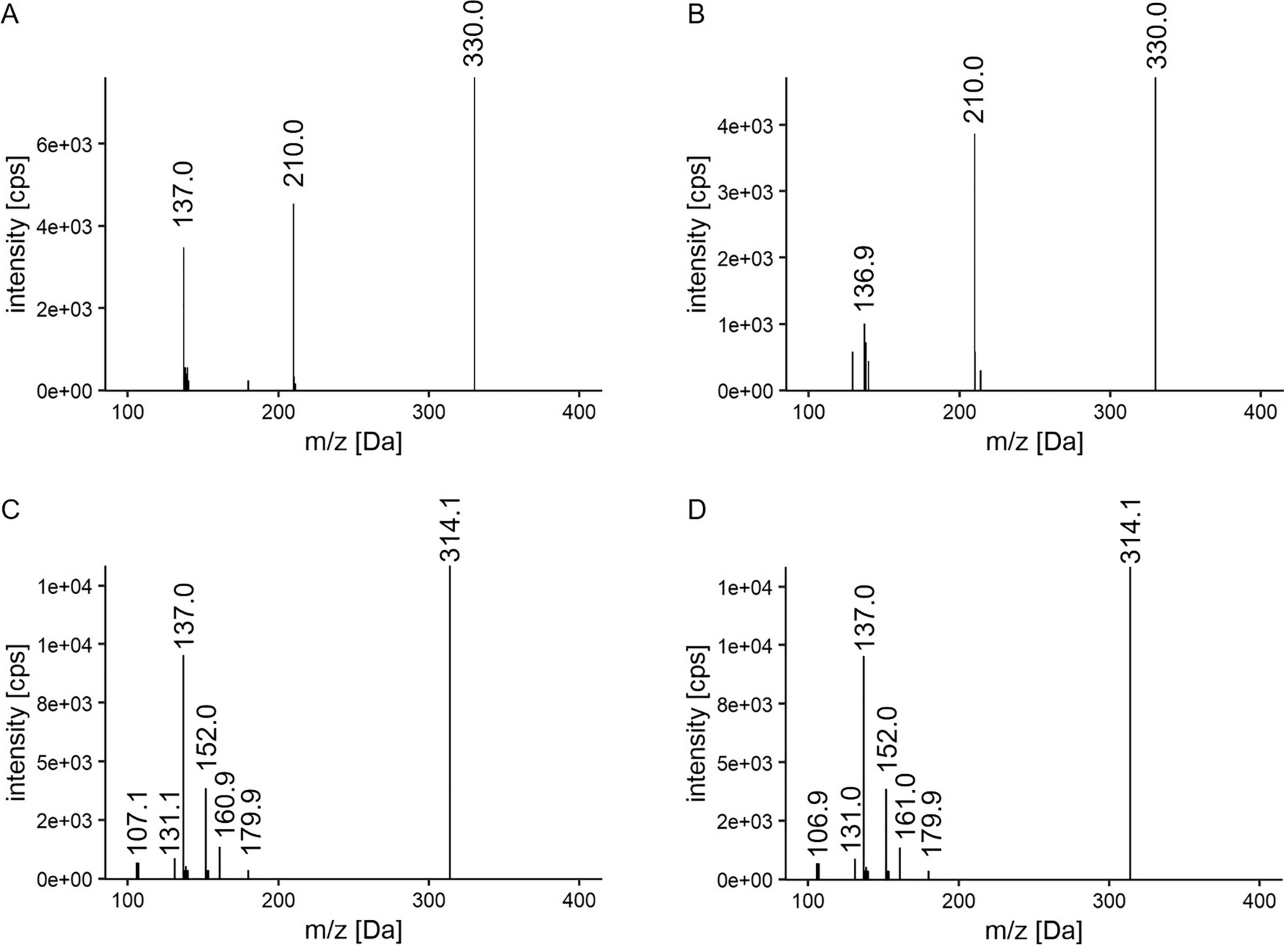
Product ion spectra of (A, C) unknown compounds in PD fluids and (B, D) the respective references (A/B) gluconic acid_3‑NPH_ and (C/D) metasaccharinic acid_3‑NPH_. The compounds were derivatized with 3-NPH prior to MS/MS analysis.

The MS data of the signals eluting at 2.1 and 4.7 min both indicated a molecular mass of 180 g/mol for the respective carboxylic acid, which is consistent with a water loss of glucose. We therefore hypothesized that these signals correspond to saccharinic acids, which are formed from glucose by rearrangement reactions.^33^ Synthesized (meta-)­saccharinic acid γ-lactone^24,25,23^ and commercial isosaccharinic acid γ-lactone were reacted with 3-NPH. The chromatographic and mass spectrometric properties of these reference compounds were compared to the unknown compounds to verify the identity of the carboxyl GDPs. Neither retention times nor product ion spectra of saccharinic and isosaccharinic acid γ-lactone derivatives matched with the data of the unknown compounds. The reference derivatives eluted at 2.6 min (saccharinic acid γ-lactone) or 2.0 min (isosaccharinic acid γ-lactone); the respective EPI spectra are displayed in the Supporting Information (Figure S1). Retention time, mass, and product ion spectra of the derivative of metasaccharinic acid γ-lactone, however, were completely consistent with the unknown compound eluting at 2.1 min (Figure 2C/D) proving the identity of metasaccharinic acid. In addition, coinjection of the metasaccharinic acid standard with a representative PD solution resulted in an increased signal at 2.1 min, without any changes in retention time or in the MS and MS/MS spectra. High-resolution mass spectrometry resulted in *m*/*z* 314.0995 ([M – H^+^]^−^, C_12_H_17_N_3_O_7_, calculated *m*/*z* 314.0983, mass error: 3.8 ppm), further confirming the identity of metasaccharinic acid.

The signal eluting at 4.7 min did not match with any of the reference compounds (Figure S2A), so that this unknown compound could not be assigned to one of the three saccharinic acids. On the basis of its mass, the compound in question might be a hitherto unknown saccharinic acid that is more hydrophobic, because it eluted more than 2 min later than the derivatives of the known saccharinic acids. However, the structure could not be assigned on the basis of the available structural information.

The signal eluting at 2.3 min was assigned to *m*/*z* 240, implying a molecular mass of 106 g/mol for the corresponding carboxylic acid. Thus, the compound could be glyceric acid, a possible breakdown product of glucose. Analysis of commercial glyceric acid under the same conditions showed that both the elution time and the EPI spectra matched those of the unknown signal (Figure 3A/B). In addition, coinjection of the glyceric acid standard with a representative PD solution resulted in an increased signal at 2.3 min, without any changes in retention time or in the MS and MS/MS spectra. High-resolution mass spectrometry resulted in *m*/*z* 240.0615 ([M – H^+^]^−^, C_9_H_11_N_3_O_5_, calculated *m*/*z* 240.0625, mass error: 4.2 ppm) and, thus, further confirmed the identification of glyceric acid in the PD fluid.

**Figure 3:**
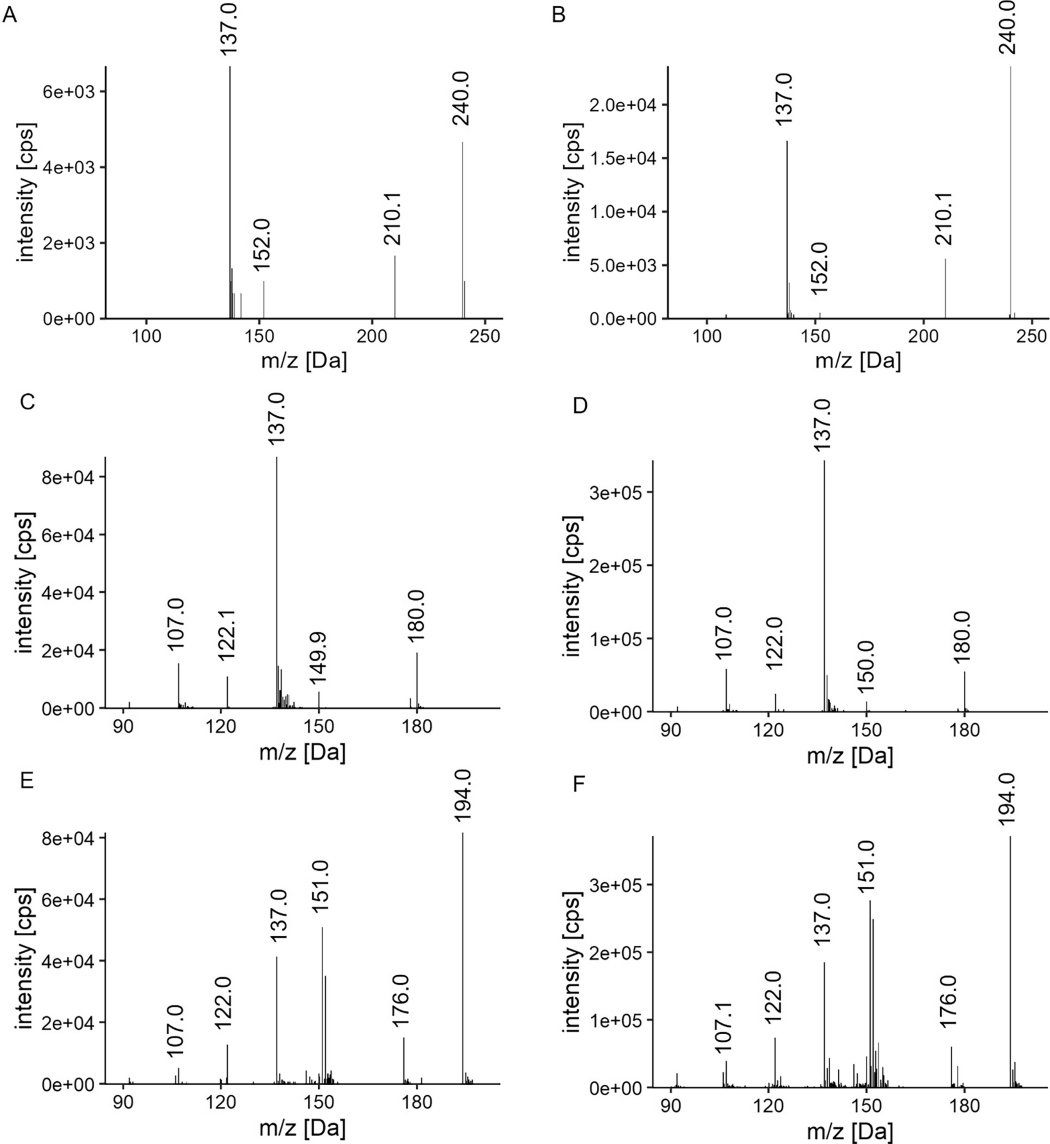
Product ion spectra of (A, C, E) unknown short-chain compounds in PD fluids and (B, D, F) the respective synthesized or commercial references (A/B) glyceric acid_3‑NPH_, (C/D) formic acid_3‑NPH_, and (E/F) acetic acid_3‑NPH_. The compounds were derivatized with 3-NPH prior to MS/MS analysis.

The signals at 3.8 and 4.4 min indicated the formation of further short-chain carboxylic acids. MS analysis revealed compounds with molecular masses of 46 and 60 g/mol, which suggested the presence of formic and acetic acid. Complete consistency of retention time and mass spectrometric data with commercial references (formic acid, Figure 3C/D; acetic acid, Figure 3E/F) proved the identity of these compounds. In addition, coinjection of the formic and acetic acid standard with a representative PD solution resulted in increased signals, without any changes in retention time or in the MS and MS/MS spectra. High-resolution mass spectrometry resulted in *m*/*z* 180.0413 for formic acid ([M – H^+^]^−^, C_7_H_7_N_3_O_3_, calculated *m*/*z* 180.0404, mass error: 5.0 ppm) and 194.0568 for acetic acid ([M – H^+^]^−^, C_8_H_9_N_3_O_3_, calculated *m*/*z* 194.0560, mass error: 4.1 ppm). The derivatization reagent blank, however, showed traces of *m*/*z* 180 and *m*/*z* 194 at the same retention times with comparable fragment spectra indicating the presence of formic acid and acetic acid in the derivatizing reagent. To rule out a false positive identification, both compounds were further analyzed by commercial enzymatic assays. This complementary method, alongside 3-NPH derivatization, confirmed the unequivocal identification of formic and acetic acid in PD fluids. The present observation is in line with previous studies, which also detected formic and acetic acid in heated glucose solutions.^34,35^

MS data for a signal at 6.8 min indicated *m*/*z* 206 (Figure S2B) and, thus, a molecular mass of 72 g/mol for the unknown acid. The signal might have referred to acrylic acid, a degradation product of glucose, but neither the chromatographic nor the MS properties were consistent with the respective reference. Therefore, a match with acrylic acid was ruled out, and this unknown structure could not be assigned.

Another putative carboxylic acid formed by the degradation of glucose was eluted at 8.5 min. The precursor mass of *m*/*z* 252 indicated a molecular mass of 118 g/mol. The product ion spectra showed just one fragment with *m*/*z* 152 in addition to the fragment *m*/*z* 137 corresponding to the hydrazide moiety of the compound (Figure S2C). To date, no structure has been proposed for this unknown compound.

Although precursor ion scans help mitigate matrix effects, the possibility of ion suppression leading to undetected novel degradation products cannot be entirely excluded. It has been reported that glucose can also degrade to dicarboxylic acids such as 2,5-furandicarboxylic acid (FDA).^22^ In addition, levulinic acid and 5-hydroxymethyl-2-furancarboxylic acid (5-HMFA) have been detected in autoclaved glucose-based solutions.^22,36^ To ensure that the precursor ion scans did not miss these compounds, targeted screening using EMS and EPI scans was carried out. Precursor masses were calculated for FDA (*m*/*z* 290 for the monohydrazide and *m*/*z* 425 for the dihydrazide) and 5-HMFA (*m*/*z* 276). Levulinic acid can form a hydrazide at C1 and a hydrazone at C4, leading to different precursors. Thus, precursor masses of *m*/*z* 250 (hydrazide) and *m*/*z* 385 (hydrazide and hydrazone) were determined. Commercial reference standards were analyzed to confirm their theoretical masses. The resulting signals were observed at retention times of 7.3 min (*m*/*z* 276) for 5-HMFA; 7.3 min (*m*/*z* 290, monohydrazide) and 10.7 min (*m*/*z* 425, dihydrazide) for FDA; and 7.0 min (*m*/*z* 250, hydrazide), 11.6 min (*m*/*z* 385, hydrazide and hydrazone), and 11.8 min (*m*/*z* 385, hydrazide and hydrazone) for levulinic acid. None of these acids could be detected in the PD fluids. Another dicarboxylic acid derived from glucose is glucaric acid. Glucaric acid, however, is only produced in the presence of very strong oxidizing agents, such as nitric acid, or through the use of catalysts.^37,38^ Since neither strong oxidizing agents nor appropriate catalysts are present in PD fluids, the formation of glucaric acid is rather unlikely.

To confirm that the signals of interest originate from heat-induced glucose degradation, a reagent blank containing water instead of PD fluid, an unheated glucose-containing PD fluid, and a glucose-free PD matrix containing the electrolytes and the buffer only were analyzed using precursor ion and enhanced mass scans. Except for formic acid and acetic acid, no comparable signals could be detected. However, the signals for formic acid and acetic acid in these blank samples were significantly lower than those in the commercial PD fluids. Therefore, it can be concluded that the signals of both acids originate from the derivatization reagent but those formic and acetic acids are also formed during heat sterilization. MRM scans were additionally performed for gluconic, metasaccharinic, and glyceric acids. No comparable signals were detected in blanks without glucose, whereas the unheated glucose-containing PD fluid exhibited comparable signals for glyceric and gluconic acids, suggesting their presence in glucose raw material. The peak intensities, however, were substantially lower compared to those of the heat-sterilized PD fluids, supporting the conclusion that these acids are formed during heat treatment. The detection of the signals in the unheated solutions by MRM but not by EMS can be explained by the higher sensitivity of the method.

As an alternative approach, hydrophilic interaction liquid chromatography (HILIC) coupled with direct detection of the acids by MS/MS without prior derivatization was considered and evaluated. However, it was found that this method lacked sufficient robustness for samples containing a large excess of glucose and lactate relative to carboxylic acids. Thus, this approach was not pursued further, and the quantification of gluconic, metasaccharinic, and glyceric was performed after derivatization with 3-NPH by LC-MS/MS in MRM mode. Because the derivatization reagent showed signals comparable to formic and acetic acids, formic and acetic acids were quantified using enzymatic assays.

To ensure correct quantification of the carboxylic acid hydrazides, the derivatization procedure was validated. First, the effect of derivatization time on hydrazide signal intensity was investigated in order to determine the optimal derivatization time. Second, the stability of the hydrazides at room temperature was tested. Signal intensities increased with derivatization time up to 60 min and remained stable thereafter across all concentration levels (signal change ≤13%). Since preliminary experiments indicated that extended derivatization times may lead to artifact formation (data not shown), a derivatization time of 60 min was chosen for subsequent analysis. Repeated analysis of both the standard solutions and the spiked PD matrix confirmed that the hydrazide derivatives remained stable for up to 6 h postderivatization (Figure S3). Thus, all samples were measured within 6 h after 60 min derivatization.

For method validation, the parameters linearity, intermediate precision (intraday), reproducibility (interday), recovery rate, LOD, and LOQ were assessed. The calibration models for gluconic, metasaccharinic, and glyceric acids showed very good linearity over the respective concentration ranges with coefficients of determination >0.9999 (Table 2). The assumption of homoscedasticity of variances was violated across the concentration range for all three acids (F-test, *p* < 0.05). Thus, weighted linear regression models were applied. The recovery rates and reproducibility were determined at three different concentration levels on three different days. In all cases, the recovery did not deviate more than 20% from the added concentration, and the reproducibility was less than 10% (Table 3). Intermediate precision (intraday) ranged from 2.4 to 11.9% for gluconic, from 1.0 to 3.2% for metasaccharinic, and from 1.0 to 7.4% for glyceric acids across all concentration levels. The results verify that the described procedure is a reliable and precise method for quantifying gluconic, metasaccharinic, and glyceric acids in PD fluids.

**Table 2:** Weighted Linear Regression Models, Limit of Detection (LOD), and Limit of Quantification (LOQ) for the Quantification of Gluconic, Metasaccharinic, and Glyceric Acids in PD Fluids

analyte	concentration range [μM]	weighted linear regression	***R*** **^2^**	LOD [μM]	LOQ [μM]
gluconic acid	1.0–30.1	*y* = 0.0786*x + 0.0141	1.0000	0.26	0.87
metasaccharinic acid	1.0–148.8	*y* = 0.2292*x + 0.0724	0.9999	0.21	0.70
glyceric acid	0.5–10.0	*y* = 0.1944*x + 0.0063	1.0000	0.03	0.11

**Table 3:** Recovery Rates and Reproducibility Expressed as the Relative Standard Deviation (RSD) of Gluconic, Metasaccharinic, and Glyceric Acids in PD Matrix

analyte	added concentration [μM]	analyzed concentration [μM]	recovery rate [%]	reproducibility RSD [%]
gluconic acid	5.2	6.2	119.3	9.5
gluconic acid	15.5	17.1	110.6	3.8
gluconic acid	25.8	26.0	100.9	5.8
metasaccharinic acid	10.1	12.1	119.8	6.7
metasaccharinic acid	50.3	58.8	115.5	5.3
metasaccharinic acid	100.7	106.6	105.8	7.4
glyceric acid	1.1	1.3	114.2	3.4
glyceric acid	3.7	3.3	88.5	4.0
glyceric acid	8.3	8.1	98.0	4.7

The newly developed UHPLC–MS/MS method was employed to monitor concentrations of gluconic, metasaccharinic, and glyceric acids, while formic and acetic acids were quantified using enzymatic assays (Table 4). Carboxylic acid concentration was determined from three measurements. In all cases, all carboxylic acids were above LOQ, indicating their formation in PD fluids.

**Table 4:** Concentration Levels of Gluconic, Metasaccharinic, Glyceric, Acetic, and Formic Acids in PD Fluids from Five Different Lots^a^

sample	gluconic acid [μM]	metasaccharinic acid [μM]	glyceric acid [μM]	acetic acid [μM]	formic acid [μM]	sum [μM]
PD fluid 1	61.6	164.1	6.6	33.0	81.1	346.4
PD fluid 2	55.2	138.1	4.8	29.1	60.1	287.3
PD fluid 3	60.8	155.0	3.8	30.7	70.4	320.7
PD fluid 4	47.8	125.8	3.2	28.4	92.3	297.5
PD fluid 5	62.6	180.7	4.6	37.3	88.1	373.3

a All values are expressed for the free acid in ready-to-use PD fluids.

Previous studies focused on the identification and analysis of α-dicarbonyl and monocarbonyl GDPs in PD fluids.^3–6,8^ For the first time, the present study focused on carboxyl GDPs and developed a targeted screening method for the application in PD fluids. Gluconic acid, metasaccharinic acid, glyceric acid, acetic acid, and formic acid, which had not been detected in PD fluids so far, could now be identified and quantified as novel GDPs. Previous studies have mainly described the formation of acids during sugar degradation under alkaline conditions,^39^ but this study demonstrates that acids can also be formed under acidic conditions. Gluconic acid usually results from the mild oxidation of glucose Scheme 2). Alternatively, it could be formed from glucosone, which is a well-documented α-dicarbonyl GDP in PD fluids,^3^ after a benzylic rearrangement.^20,40^ Free gluconic acid is in equilibrium with γ-lactone and δ-lactone in aqueous solutions but predominates at pH 3 and lower. PD fluids are buffered with lactate to pH 5.0–5.5. Therefore, it can be assumed that free gluconic acid is present in PD fluids in equilibrium with the γ-lactone and δ-lactone. To investigate whether lactones and the free acid react with 3-NPH in a similar manner, glucono-δ-lactone and potassium gluconate were reacted with 3-NPH. Both forms gave the expected signal at a retention time of 1.6 min. Thus, the screening method was able to detect gluconic acid independent from the acid’s form.

**Scheme 2:**
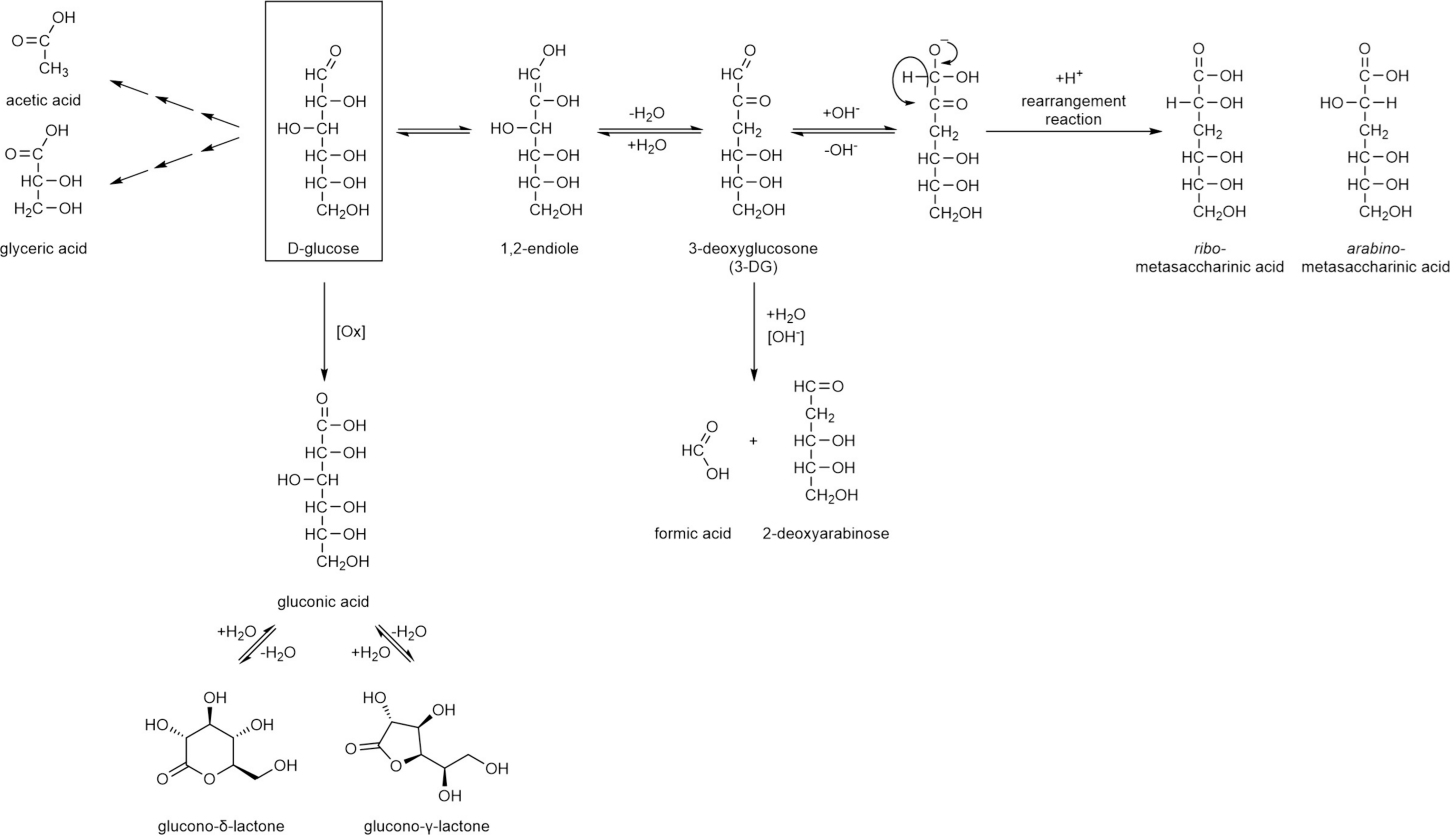
Proposed Formation Pathways of Carboxylic Acids in PD Fluids during Glucose Degradation

α-Dicarbonyl GDPs such as 1-DG, 3-DG, or 4-DG can react to saccharinic acids.^33,41^ Both Nef and Isbell suggested that the α-dicarbonyl compounds undergo a type of benzylic acid rearrangement to form saccharinic acids (Scheme 2).^20,40,42^ This base-catalyzed reaction, however, requires higher pH values.^21^ Presently, metasaccharinic acids were also formed in heat-sterilized PD fluids at lower pH values. The precursor of metasaccharinic acid is 3-DG, one of the major GDPs in glucose-based PD fluids,^3^ while 1-DG can be degraded to saccharinic acid^41^ and 4-DG to isosaccharinic acid.^43^ The fact that no relevant concentrations of 1-DG or 4-DG have been detected as yet in glucose-containing PD fluids^3^ is probably the reason why these degradation products could not be detected in the present study.

3-DG may not serve as a precursor for metasaccharinic acid only but also for formic acid by C1–C2 cleavage (Scheme 2).^35^ Another formation pathway for formic acid has been described via 5-HMF, which can be degraded to formic acid and levulinic acid at a molar ratio of 1:1.^36^ Since no levulinic acid could be detected in the presently investigated PD fluids, the formation of formic acid via 5-HMF may play a minor role in PD fluids.

Glyceric acid may result from the degradation of 1-DG through different pathways. One pathway involves a nucleophilic attack at the C4 position of the isomeric 1-deoxyhexo-2,4-diulose, while another one includes the oxidation of the isomeric 1-deoxyhexo-3,4-diulose, followed by C3–C4 cleavage.^35,44^ Another proposed formation pathway suggests the oxidation of 1-DG to a tricarbonyl compound, which then undergoes β-cleavage at the C3–C4 bond.^41,44^ In addition, 1-DG has been described as a precursor of acetic acid by C2–C3 cleavage.^35,45^ The presence of 1-DG, however, has not been reported yet in PD fluids.^3^ It is possible that 1-DG is rapidly degraded into compounds such as acetic acid or glyceric acid so that its concentration is below the detection/quantification limit of the current analytical methods. Alternatively, as yet unknown formation pathways might significantly contribute to the contents of glyceric and acetic acid in PD fluids.

The most abundant carboxylic acid was metasaccharinic acid with concentration levels up to 180.7 μM, followed by formic (up to 92.3 μM), gluconic (up to 62.6 μM), acetic (up to 37.3 μM), and glyceric acid (up to 6.6 μM). The total amount of carboxylic acids ranged from 287.3 to 373.3 μM. The newly identified carboxyl GDPs were detected at concentration ranges comparable to those previously reported for monocarbonyl and α-dicarbonyl GDPs.^3^ For example, 3-deoxyglucosone, currently recognized as the most abundant α-dicarbonyl GDP in glucose-containing PD fluids, has been detected at concentrations ranging from 236 to 256 μM.^8,46^ Glyoxal and methylglyoxal have been detected with concentrations between 4.6–8.3 and 7.9–9.3 μM.^5,8^ 5-HMF, a common monocarbonyl GDP, has been reported at concentrations of 11.1–12.7 μM in comparable PD fluids.^5,6^ This demonstrates that the newly identified carboxyl GDPs occur at concentrations comparable to, or even exceeding, those of well-established GDPs with monocarbonyl or α-dicarbonyl structure.

The calculated pH values after sterilization, based on the newly formed acids, closely matched the measured pH values (Table 5). The measured and calculated pH values differed by no more than 0.05, except for one sample, where the difference was 0.11. This suggests that the observed pH decrease during sterilization can be mainly explained by the formation of gluconic, metasaccharinic, glyceric, acetic, and formic acids; consequently, the precursor ion approach appears to have captured the major carboxylic acids in the samples analyzed.

**Table 5:** Measured and Calculated pH Values after Sterilization of Lactate-Buffered PD Fluids^a^

sample	measured pH poststerilization	calculated pH poststerilization
PD fluid 1	5.30	5.34
PD fluid 2	5.35	5.37
PD fluid 3	5.32	5.35
PD fluid 4	5.25	5.36
PD fluid 5	5.32	5.33

a Calculated pH values derived from a proton balance model based on acid dissociation of measured carboxyl GDPs and buffer capacity.

For short-chain organic acids, early *in vitro* studies described cytotoxicity as being predominantly mediated by pH-dependent proton influx and subsequent intracellular acidification.^47^ In addition to this general mechanism, more recent studies have shown substance-specific cytotoxic effects for formic acid and its dissociated form, formate. In particular, the involvement of apoptotic signaling pathways has been reported and a concentration- and time-dependent cytotoxicity of sodium formate has been demonstrated in HT22 neuronal cells.^48^ Although the concentrations used in this study (12.5–100 mM) exceeded those in PD fluids, formate and formic acid can accumulate *in vivo* under certain conditions.^49^ Thus, cumulative exposure to low concentrations of formic acid/formate may contribute to toxic effects. This may be particularly relevant in the context of PD, where PD fluids are administered repeatedly and local accumulation may therefore be considered.

The newly identified and quantified compounds have not been reported in PD fluids to date, largely because previous analytical methods did not cover carboxylic acids. This study introduces an approach that enables the detection of previously overlooked degradation products in PD fluids and provides a more comprehensive characterization of the GDP profiles. It highlights the value of advanced analytical strategies for the detailed analysis of PD fluids.

## Conclusions

The present identification and quantification of gluconic, metasaccharinic, glyceric, acetic, and formic acids as novel GDPs provide an explanation for the observed drop in pH during the heat sterilization of glucose-containing PD fluids. In addition, it was also shown that glucose degradation at acidic pH values can lead to the formation of carboxylic acids. How degradation products influence the biocompatibility of PD fluids can only be assessed when potential GDP structures and concentration levels are fully elucidated. The present study contributes to the knowledge on carboxyl GDPs. In the next step, the biological and clinical relevance of the new carboxyl GDPs can be determined, and strategies can be developed to decrease the formation of clinically relevant GDPs in PD fluids.

## Supplementary Material



## Notes

Figure S1, Product ion spectra of saccharinic acid γ-lactone and isosaccharinic acid γ-lactone; Figure S2, Product ion spectra of unknown compounds in PD fluids; Figure S3, Time-dependent stability of derivatized standard solutions and standards in PD matrix; Table S1, Multiple reaction monitoring transitions and compound-specific mass spectrometry parameters used for the quantification of gluconic, metasaccharinic, and glyceric acids in PD fluids (PDF)
