# Supplementary material for: Identification and Quantification of Gluconic, Metasaccharinic, Glyceric, Formic, and Acetic Acids as Novel Glucose Degradation Products in Peritoneal Dialysis Fluids
Source: ACS Omega. 2026 Jun 4;11(23):34338–49. doi: 10.1021/acsomega.6c02048 (PMC13280894; doi:10.1021/acsomega.6c02048)
Supplement: Supplementary file 1 [file ao6c02048_si_001.pdf]

Supporting Information

Identification and Quantification of Gluconic, Metasaccharinic, Glyceric, Formic, and Acetic Acids as Novel Glucose Degradation Products in Peritoneal Dialysis Fluids

Sabrina Gensberger-Reigl[1,2]*, *Jinan Awada*[1], *Vera L. Rodrigues Guimarães Abreu*[1], *Ingrid Weigel*[1], *Dimitra Tousi*[1], *Elisa Gardill*[1,3], *Pascal Mathis*[3], *Monika Pischetsrieder*[1,2]

[1]Chair of Food Chemistry, Department Chemistry and Pharmacy, Friedrich-Alexander-Universität Erlangen-Nürnberg (FAU), Nikolaus-Fiebiger-Str. 10, 91058 Erlangen, Germany

[2]Fresenius Medical Care Deutschland GmbH, Frankfurter Straße 6-8, 66606 St. Wendel, Germany

[3]FAU NeW - Research Center New Bioactive Compounds, Friedrich-Alexander-Universität Erlangen-Nürnberg (FAU), Nikolaus-Fiebiger-Str. 10, 91058 Erlangen, Germany

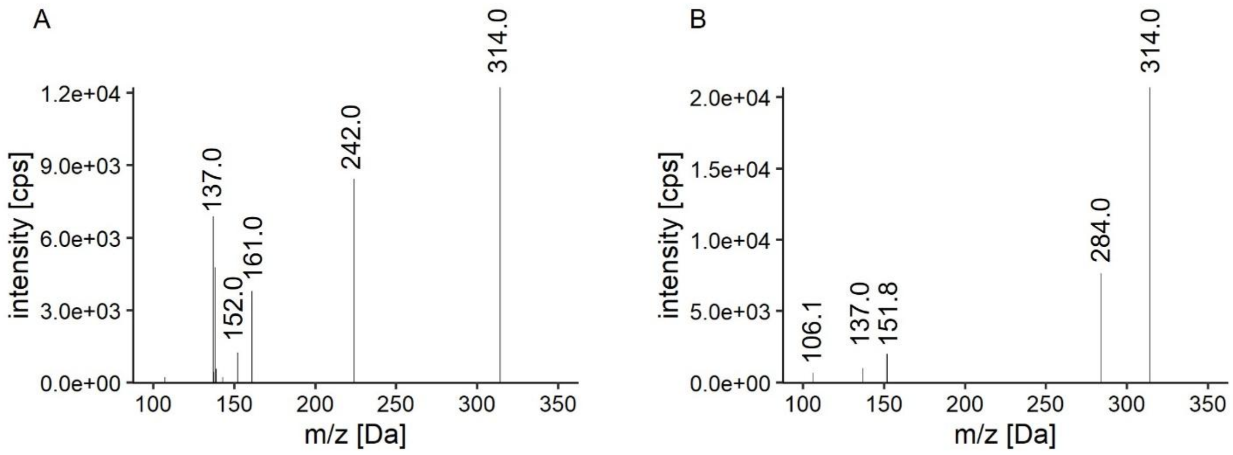

Figure S1 Product ion spectra of (A) saccharinic acid γ-lactone and (B) isosaccharinic acid γ-lactone. The compounds were derivatized with 3-NPH prior to MS/MS analysis.

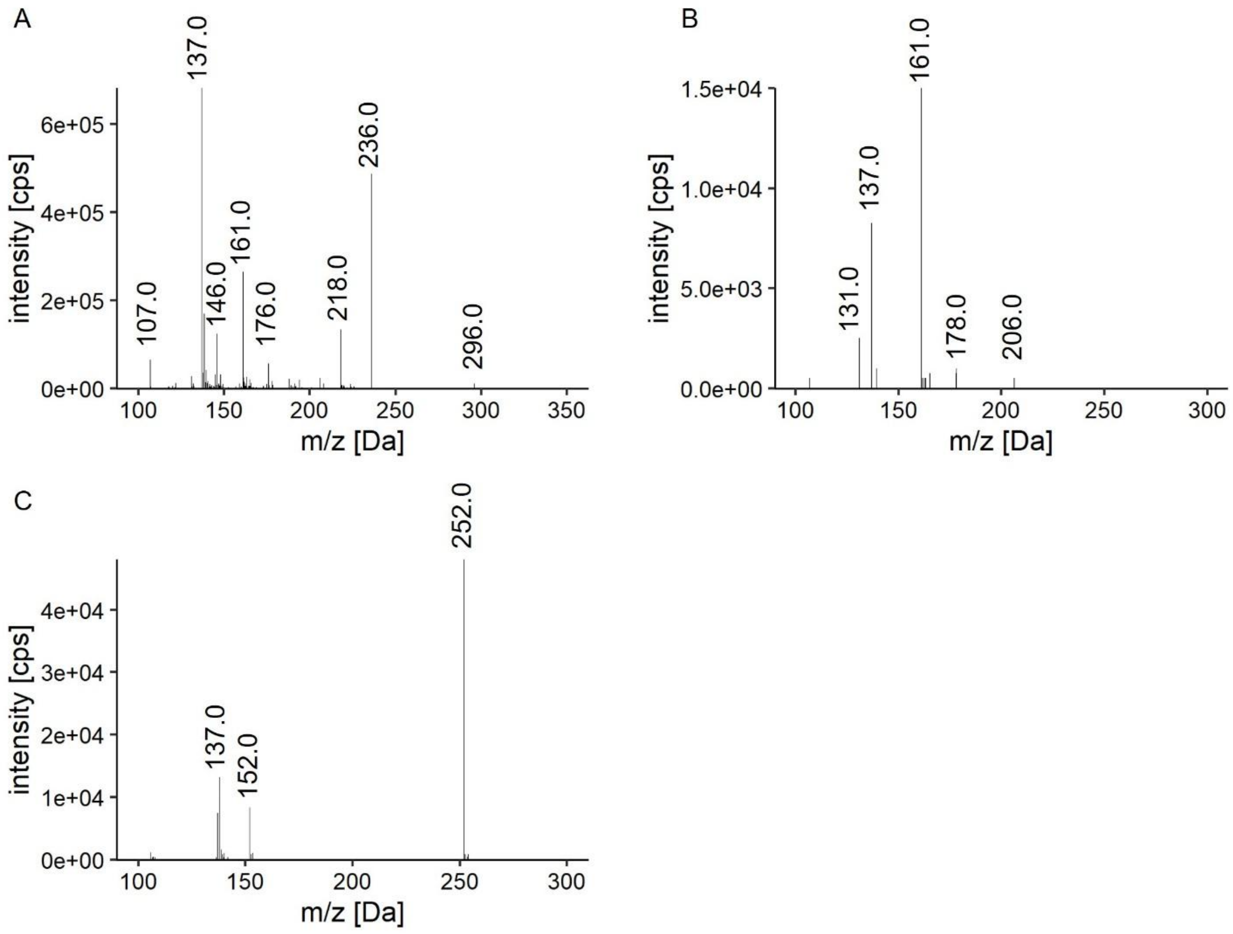

Figure S2 Product ion spectra of unknown compounds in PD fluids. The compounds were derivatized with 3-NPH prior to MS/MS analysis. (A) compound m/z 314, (B) compound m/z 206, (C) compound m/z 252.

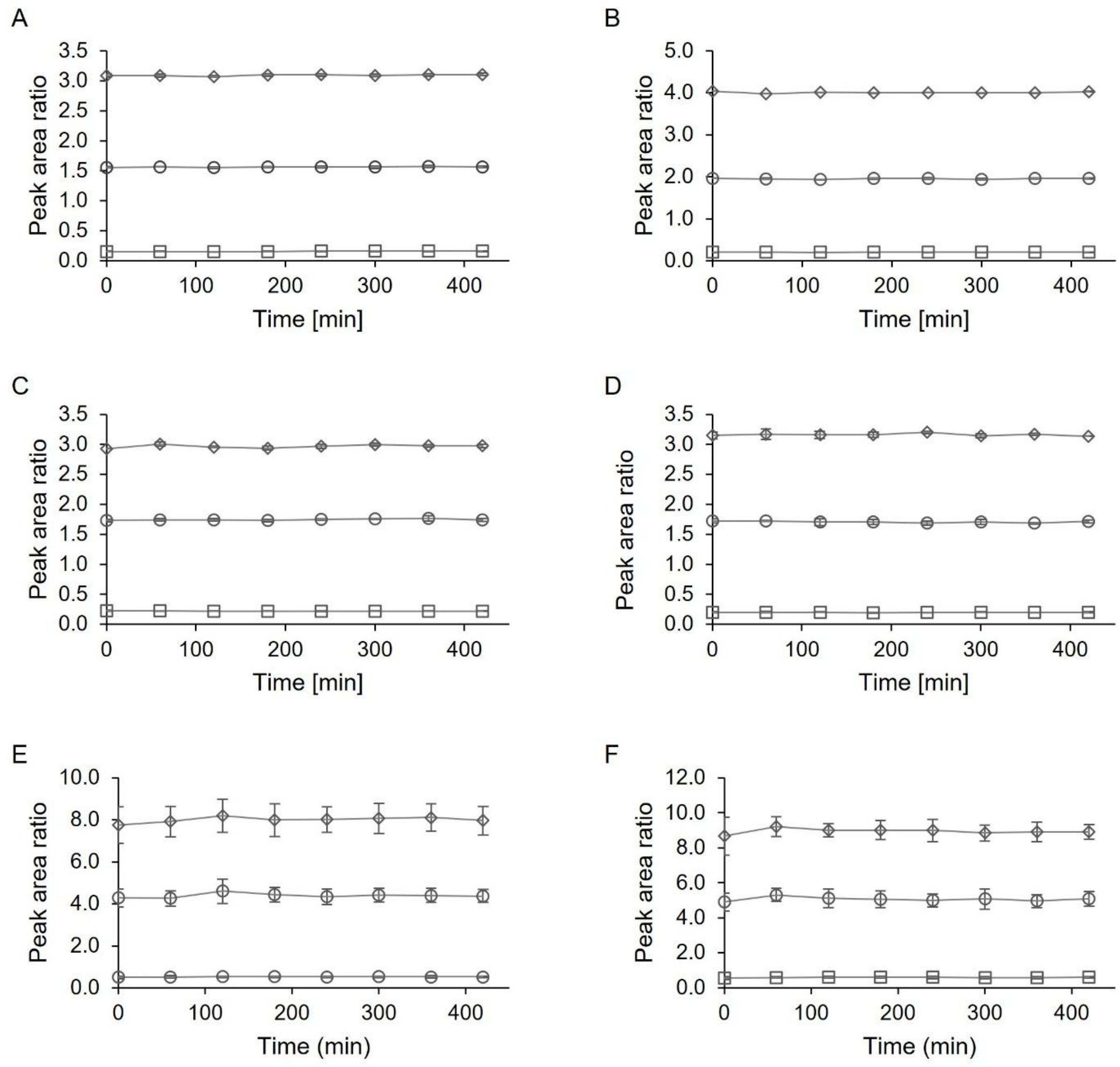

Figure S3 Time-dependent stability of derivatized standard solutions (A, glyceric acid; C, gluconic acid; E, metasaccharinic acid) and standards in PD matrix (B, glyceric acid; D, gluconic acid; F, metasaccharinic acid) at three different concentration levels (◊ 200 µM; ○100 µM; □ 10 µM). The peak area ratio between the standard compound and its respective isotopically labelled internal standard is plotted as a function of time between the end of derivatization and analysis. ^{13}C-gluconic acid was used as isotopically labelled standard for metasaccharinic acid.

Table S1 Multiple reaction monitoring transitions and compound-specific mass spectrometry parameters used for the quantification of gluconic, metasaccharinic, and glyceric acids in PD fluids. Transitions marked with an asterisk (*) were used as quantifiers, while the remaining transitions served as qualifiers.

**compound**	**precursor *m/z***	**fragment *m/z***	**declustering potential [V]**	**collision energy [eV]**	**cell entrance potential [V]**	**cell exit potential [V]**
gluconic acid	330.01*	152.08*	−50	−35	−5	−11
	330.01	129.08	−50	−20	−10	−5
	330.01	194.01	−50	−30	−10	−8
gluconic acid-1-^{13}C	331.10*	152.02*	−60	−35	−12	−8
	331.10	223.05	−50	−25	−12	−11
	331.10	129.98	−50	−25	−12	−11
metasaccharinic acid	314.12*	161.03*	−50	−25	−10	−11
	314.12	180.00	−60	−30	−10	−17
	314.12	206.02	−50	−30	−12	−17
glyceric acid	240.09*	210.07*	−60	−20	−12	−8
	240.09	180.01	−60	−20	−12	−5
	240.09	121.99	−60	−30	−5	−11
^{13}C-glyceric acid	243.08*	212.03*	−70	−20	−8	−11
	243.08	122.06	−70	−30	−10	−8
	243.08	91.97	−60	−40	−10	−11
